# Should peanut allergy screening be introduced for all Irish children?

**DOI:** 10.1186/2045-7022-5-S3-P13

**Published:** 2015-03-30

**Authors:** Maeve Kelleher, Claire Culliane, Audrey Dunn Galvin, Deirdre Murray, Jonathan O B Hourihane

**Affiliations:** 1 Department of Pediatrics and Child Health, University College Cork, Cork, Ireland

## Rationale

There has been an incremental increase in peanut allergy worldwide in recent decades. Unlike other major food allergens, peanut allergic children are unlikely to acquire tolerance in childhood. Peanut and treenuts are the most common foods implicated in food induced anaphylaxis. We use the data gathered from a large unselected Irish birth cohort study to assess whether screening for Peanut Allergy should be implemented in Irish children.

## Methods

2137 infants were enrolled on the BASELINE study from July 2008 to October 2012. From birth to 2 years children had 5 study appointments. Screening questions for food allergy were asked at each visit. Skin prick testing (SPT) to peanut was performed in infants who had symptoms suggestive of peanut allergy or in infants with known Egg or Cow's milk allergy. At 2 years screening for food allergy and sensitisation was offered to all participants by SPT to a panel of common food allergens including peanut. A SPT >3mm was deemed positive. Infants with peanut positive SPT not regularly and safely ingesting peanut had Oral Food Challenge (OFC) to differentiate Peanut Allergy from asymptomatic food sensitisation.

## Results

Of all 2137 infants enrolled from on to the study from July 2008 to October 2012, 1537 attended at 2 years. 1421 infants had a full food panel SPT undertaken. Peanut sensitisation rate was 2.68% (38/1421) and of these children 63% (24/38) had proven peanut allergy. Peanut allergy prevalence at 2 years was 1.69% (24/1421).

The mode of presentation of these 24 Peanut Allergic children was examined. 11 infants were confirmed peanut allergic prior to 2 years through screening when presenting with other food allergies. At 2 years 26% (11/42) of children with previously confirmed food allergy, were peanut allergic. Six infants reported an adverse reaction to peanut between 1-2 years and all 6 were confirmed peanut allergic. Just 7 infants or 0.5% (7/1373) of the remainder of the asymptomatic population were confirmed peanut allergic.

## Conclusion

The prevalence of Peanut allergy in Irish 2 year olds is 1.69%. One third of children with peanut positive testing at two years can safely tolerate peanut. It is incumbent to avoid peanut testing on asymptomatic children unless prompt access to OFC is available to differentiate between Peanut sensitization and peanut allergy. This study would suggest targeted screening of infants presenting with early onset Cow's Milk or Egg allergy would be the most appropriate use of peanut allergy screening resources.

